# The Impact of a Gamified Mobile Mental Health App (eQuoo) on Resilience and Mental Health in a Student Population: Large-Scale Randomized Controlled Trial

**DOI:** 10.2196/47285

**Published:** 2023-07-21

**Authors:** Silja Litvin, Rob Saunders, Philip Jefferies, Hayley Seely, Patrick Pössel, Stefan Lüttke

**Affiliations:** 1 Department Psychologie Ludwig-Maximilians-Universität München Germany; 2 Division of Psychology and Language Sciences Clinical Education and Health Psychology University College London London United Kingdom; 3 Resilience Research Centre Faculty of Health Dalhousie University Halifax, NS Canada; 4 Department of Counseling and Human Development College of Education and Human Development University of Louisville Kentucky, KY United States; 5 Department für Klinische Psychologie und Psychotherapie Institute für Psychologie Universität Greifswald Greifswald Germany

**Keywords:** mobile health, mHealth, gamification, resilience, randomized controlled trial, RCT, mental health, apps, mobile health, mobile game, mobile games, serious game, depression, anxiety, university, college, student, students, controlled trial, controlled trials, young adult, mobile phone

## Abstract

**Background:**

With many digital mental health interventions failing to engage clients for enough time to demonstrate substantive changes to their well-being and with only 2% of all digital solutions on app stores having undergone randomized controlled trials, the rising demand for mental health prevention and early intervention care is not being met. Young adults in particular struggle to find digital well-being apps that suit their needs.

**Objective:**

This study explored the effects of eQuoo, an evidence-based mental health game that teaches psychological skills through gamification, on resilience, depression, anxiety, and attrition in a student population.

**Methods:**

In total, 1165 students from 180 universities in the United Kingdom participated in a 5-week, 3-armed randomized controlled trial. Participants were randomly allocated into 1 of 3 groups: eQuoo users, users of a treatment-as-usual evidence-based cognitive behavioral health app called Sanvello, and a no-intervention waitlist. The Rugged Resilience Scale, Generalized Anxiety Disorder–7, and Patient Health Questionnaire–8 were administered to all participants at baseline and every 7 days until completion.

**Results:**

A repeated measures–ANOVA revealed statistically significant increases in resilience scores in the test group (*P*<.001) compared with both control groups (Sanvello: *P*=.10 and waitlist: *P*=.82) over 5 weeks. The app also significantly decreased anxiety and depression scores (both *P*<.001). With 64.5% (251/389) adherence, the eQuoo group retained 42% more participants than the control groups.

**Conclusions:**

Digital health interventions such as eQuoo are effective, scalable, and low-cost solutions for supporting young adults and are available on all leading mobile platforms. Further investigation could clarify the extent to which specific elements of the eQuoo app (including gamification) led to better outcomes.

**Trial Registration:**

German Clinical Trials Register (DRKS) DRKS00027638; https://drks.de/search/en/trial/DRKS00027638

## Introduction

### Background

Digital health interventions (DHIs) have repeatedly been shown to be effective in improving mental well-being and relieving the symptoms of depression [1-4] and anxiety [1-3,5,6] in young people. They are efficacious in young people exhibiting elevated symptoms of anxiety and depression as well as in those who have been diagnosed with an anxiety or depressive disorder [2]. Randomized controlled trials (RCTs) have also revealed that DHIs can improve mood and promote emotional resilience in adolescents in the general populations [7,8].

DHIs may have other potential benefits for adolescents and young adults. For example, even when barriers to health systems are low (eg, health insurance coverage or free health care services) and individuals report elevated depression or anxiety symptoms, the rates of engagement with mental health support are low [9,10]. This suggests that there may be further obstacles, including attitudinal barriers, such as a preference for self-reliance and the desire to avoid perceived stigmatization [9-12], and off-putting aspects related to the delivery of the service, such as physical access issues [11], in addition to long waitlist times [12,13]. DHIs may help address these barriers, as research has shown that young people are drawn to digital health solutions [14,15], especially those on mobile phones [16].

DHIs delivered via mobile phones present a promising route for supporting the mental health of young people. For example, the ownership of mobile phones has grown enormously in recent years, with a national US survey finding that in 2019, approximately 90% of 16- to 18-year-olds possessed a smartphone, compared with 75% in 2015 [17]. There is also a high motivation to use mobile phone apps to improve mental health [18], which may stem from not only their convenience but also their control in terms of when and how much an individual chooses to engage with a DHI.

DHIs delivered by mobile phones typically take the form of “unguided” apps. Although numerous systematic reviews have found that DHIs for young people were effective when they incorporated some sort of human support [4,6,9,19], such as professional feedback or a live link to a professional, their superiority over unguided DHI approaches [20] has not been confirmed conclusively. For instance, in some studies, the effect in favor of guided DHIs was small [20] or not statistically significant when only studies with a low risk of bias were considered [19]. For smartphone-based interventions for depressive symptoms, a recent meta-analysis of RCTs by Firth et al [21] found that only apps without professional feedback produced moderate positive effects. Another meta-analysis found larger effect sizes on stress levels and quality of life when apps provided professional guidance [22].

Despite the convenience, appeal, and promise of DHIs delivered by mobile phones, evidence for their efficacy remains unsatisfactory. For example, review studies can neglect the modality of a DHI, which is important when the current bulk of studies involve comparisons of guided internet-based or computer-based cognitive behavioral therapy (CBT) with inactive controls [2,23]. Furthermore, in a recent review of 19 studies that investigated the use of serious games and virtual reality for the treatment of common mental health problems in children and young people, 10 (53%) had no comparison group, 3 (16%) used a waitlist condition, 4 (21%) used another DHI control group condition, and only 2 (11%; investigating the same DHI) used face-to-face interventions [3,4]. The effect of DHIs was largest when DHIs were compared with nonactive control conditions and was small or even nonexistent when there was an active comparator [4,22,23]. Recent meta-analyses that have specifically explored studies involving mobile phone apps for depression and anxiety have suggested that they have the potential to reduce symptoms when compared with inactive controls [21,24], and a recent meta-review by Goldberg et al [25] supports these findings. The authors noted that there are few studies involving active controls, which is important for establishing the effect of using a particular intervention.

A further limitation affecting recent studies on the efficacy of DHIs on mental health in young people (including DHIs delivered via mobile phones) was highlighted by Grist et al [4] in their meta-analysis of the efficacy of DHIs for depression and anxiety in children and adolescents. The authors found that many of the studies were underpowered. Insufficient sample sizes can promote favorable effects of particular interventions [1,2,5], reducing the likelihood that a statistically significant result reflects a true effect [26,27].

Aside from evaluation issues, a further issue troubles DHI research. In 2005, Eysenbach [28] noted that a considerable number of study participants stopped engaging with them (ie, nonuse attrition or nonadherence) or dropped out; therefore, they could not be followed up (dropout attrition). For example, Linardon et al [22] found that approximately 25% of participants using smartphone-delivered DHIs for common mental health problems dropped out before short-term follow-up (≤8 weeks) and up to 33% dropped out before long-term follow-ups. Similarly, Hollis [23] found that for DHIs targeting depression in young people, attrition ranged from 0% for a computerized in-house attention bias modification [29] to 42.5% for a synchronous chat intervention [30]. Although this is potentially problematic for evaluating the efficacy of DHIs, the lack of or diminishing engagement is troubling, given the benefits of engaging with such interventions. Developers of non–practitioner-led (unguided) DHIs face an overarching challenge: how to engage clients long enough for interventions to have an impact.

One approach to combating attrition and nonengagement in DHI design is the use of game design elements in nongaming contexts or “gamification” [31]. Although the gamification of software and technology in a behavior change context has gained traction over the past few years [32], it is still in the early stages in terms of methodology, classification, and implementation. In total, 2 relevant research streams have emerged: (1) persuasive systems and technology and (2) gamification as a tool that enables playful experiences and enhances engagement [33]. Persuasive systems are geared toward using software and technology to instigate behavioral change regarding preset goals, such as weight loss, developing beneficial psychological coping mechanisms, or even environmental sustainability actions [34]. Persuasive strategies can be implemented endogenously; software can be developed via game mechanics, which are intrinsically inherent to the gaming experience [35]. Endogenous games are designed to enhance the gaming experience to the highest degree possible; external goals of persuasive systems, where game mechanics rules are coupled with outcome goals, are likely to hinder or complicate the process. This is sometimes referred to as gamifying rather than gamification. The more commonly used gamification development procedure for persuasive games, where elements are layered on an existing framework of information exchange geared toward a predefined goal (such as weight loss), is exogenous [35]. Although it makes sense to implement evidence-based strategies, such as CBT, as the foundation of a persuasive system for a DHI, it can be at odds with the second research stream: playful experiences and enhanced engagement. Poorly implemented exogenous factors, such as badges or rewards that are not connected to the gameplay, have reportedly had the opposite effect on the playing population, leading to the rejection of the tool [36]. An example of an endogenous game would be eQuoo, where the intervention is woven into a story framework that is appealing in and of itself, whereas an exogenous element would be a badge given after a CBT exercise is successfully completed.

In addition to gamification, interventions aimed at building skills associated with improved mental health may help enhance mental health overall. As conceptualizations of mental health expand beyond the pathogenic to the salutogenic [37], researchers have started to investigate mental health in terms of both the capacity to manage difficulties and the absence of illness [38]. Resilience is widely considered to be a protective factor against negative mental health outcomes. Resilience is the capacity to overcome or adapt to adversity and thus stay mentally healthy or regain one’s mental health following significant challenges [39]. Previously thought to reflect the presence of personality characteristics, such as *grit*, resilience is now more commonly understood as a process in which various modifiable protective factors are drawn upon. This leads to positive outcome trajectories [40]. Protective factors exist at various systemic levels, including psychological (eg, self-efficacy and motivation) and social (eg, supportive peer relationships and a sense of community belonging) [41]. The realization that many individuals experience significant adversity during their lifetime [42] has led to an increasing demand for resilience-building programs aimed at promoting mental health [34].

### This Study

In summary, research has shown that DHIs can be effective in promoting mental well-being and reducing symptoms of depression and anxiety in young people. However, the evidence base is lacking, and given their unique qualities, there is a need to specifically test the advantages of using DHIs involving gamification and through sufficiently powered 3-armed RCTs (DHIs vs active controls vs nonactive controls) that also report on trial completion and attrition.

#### Primary Outcome Hypothesis—Resilience Levels

We hypothesized that resilience levels would increase significantly over the course of the intervention period in the gamified intervention group and that resilience would be significantly higher in the intervention group than in the active control and waitlist control groups.

#### Secondary Outcome Hypotheses—Depressive and Anxiety Symptoms and Attrition

We also hypothesized that depression and anxiety symptoms would decrease significantly over the course of the intervention period in the gamified intervention group and that depression and anxiety symptoms would be reduced significantly in the gamified intervention group compared with both the active control and waitlist control groups.

Finally, we hypothesized that rates of attrition would be significantly lower in the gamified intervention group at the last assessment than in the active control and waitlist control groups.

## Methods

### Inclusion and Exclusion

Participants had to be aged ≥18 years and enrolled as college or university students. They had to have access to a smartphone or tablet device and an app store (App Store or Google Play) to install the eQuoo app. No further exclusion criteria were applied.

### Sampling Procedure and Participant Consent

Recruitment took place in 2 waves between March and April 2021 using the UNiDAYS subscriber database, a global student discount platform. To qualify for UNiDAYS, people must provide a valid college or university email address. In the first wave, 9000 people who were then registered on the platform with their University College London affiliation were emailed an invitation to participate in a trial exploring the effects of a mental health app on resilience with a link to the trial landing page; 443 people signed up for the trial and completed the baseline assessment. This figure represented 8.6% of those emailed. On the landing page, the study goal was explained briefly, the study protocol was shared, and a consent box needed to be clicked that read “I have understood the trial terms and I consent.” This checkbox needed to be checked for participants to join the trial; without it, they could not gain access to the questionnaires and information on what to test, thus ensuring consent. In the second wave, 100,000 randomly selected UNiDAYS subscribers were emailed the same invitation; 724 participants completed the baseline assessment. This figure represented 1.6% of those emailed. The trial was completed and closed, and all participants were debriefed in June 2021.

### Ethics Approval

The study (an RCT) was approved by the University College London Ethics Committee (0501/001), and the authors confirm that all ongoing and related trials are registered with the German Clinical Trials Register (DRKS00027638). This study was conducted in accordance with the principles of the Declaration of Helsinki.

### Sample Size, Power, and Precision

An a priori power analysis using G*Power 3.1.9.4 revealed that a sample size of 207 participants was required to detect a within-between interaction for the primary outcome (input parameters: repeated measures [RM]–ANOVA, effect size *f*=0.10; Cronbach α=.05; power=0.95; number of groups=3; number of measurements=6; RM intercorrelation=0.50; nonsphericity correction=1). However, given the high attrition rates reported in previous research (eg, 40%-60% [28]), we aimed to enroll a minimum of 850 participants.

### Measures and Instruments

#### Primary Outcome: Resilience

As eQuoo exists primarily in the digital space of prevention and early intervention, resilience was considered the primary outcome. To assess resilience, we used the Rugged Resilience Measure (RRM). The RRM is a 10-item self-report questionnaire designed to measure key psychologically protective factors that foster resilience [43]. Participants respond to the items on a 5-point Likert-type scale (1=*not at all*, 2=*a little*, 3=*somewhat*, 4=*quite a bit*, and 5=*a lot*). The questionnaire was initially validated with a sample of young adults (aged 16-30 years), which matched the population of the study. As the understanding of resilience has shifted from a fixed trait to a process encompassing the development and application of skills and resources that support positive outcomes despite the experience of distress [44,45], the RRM taps the key internal resources necessary to initiate said development. In this study, the internal consistency of the RRM was .87 (Cronbach α).

#### Secondary Outcomes: Anxiety, Depression, and Attrition

The secondary outcomes in this study were mental health (the level of anxiety and depression symptoms in particular) and attrition. The generalized anxiety disorder–7 (GAD-7)-item scale was used to assess anxiety. This is a widely applied 7-item measure of generalized anxiety symptoms [46]. Each item was scored from 0 to 3 (0=*not* *at all*, 1=*several days*, 2=*more than half the days*, and 3=*nearly every day*). The internal consistency of the GAD-7 was high (Cronbach α=.83). We also used the Patient Health Questionnaire–8 (PHQ-8). The PHQ-8 is a well-established screening measure for depressive symptoms in large clinical studies [47] and encompasses the American Psychological Association’s *Diagnostic and Statistical Manual of Mental Disorders* (5th edition) criteria for a depressive episode (with the exception of the item regarding self-harm). It was preferred to the more widely known PHQ-9 [27] because the study design did not allow us to intervene in the event of reporting self-injurious behavior. In the PHQ-8, participants are asked to rate how far they have been bothered by each symptom over the previous 2 weeks on a 4-point Likert-type scale (0=*not at all*, 1=*several days*, 2=*more than half the days*, and 3=*nearly every day*). The internal consistency of the PHQ-8 in this study was high (Cronbach α=.88). Finally, it was hypothesized that attrition would be significantly lower for the gamified group intervention at the last assessment (t5) than for other groups. In addition to measures of resilience, anxiety, and depression, participants provided demographic information regarding their age, sex, living situation, and level of study.

#### Design

A mixed factorial 3 (condition) × 6 (time) RM design was used. Participants were randomly assigned to a condition (eQuoo vs active control vs waitlist group, the details of which are described in the subsequent section) using a randomization generator provided by random.org [48], which randomizes based on atmospheric noise [49]. Across the study period, measurements were taken at the beginning (t0, baseline assessment), week 1 (t1), week 2 (t2), week 3 (t3), week 4 (t4), and week 5 (t5, end point).

#### Gamified Group Intervention (eQuoo)

The original version of the emotional fitness app eQuoo was a 5-week mental health game that presented psychoeducational material and psychological exercises based on the principles of CBT, systemic psychology, and positive psychology [50]. It combined a mix of endogenous and exogenous design features designed to maximize engagement. The game was played on mobile phone only and was available internationally on the Google Play store and the Apple App Store. It was designed to be a prevention and early intervention (preclinical threshold) tool. In a recent trial, the app was found to significantly reduce anxiety while improving resilience, perceived growth skills, and interpersonal relationships [51]. After examining feedback from thousands of users through clinical trials, focus groups, case studies, and players offering their opinions on the app stores and via email, the developers of eQuoo sought to revise the app to enhance enjoyment, immersion, and retention. This resulted in eQuoo, the Next Generation [50] (hereafter, eQuoo), and it is the version of the app that was tested in this study.

The app begins by explaining the player’s role as a Lodestar. Each page has a picture with characters in it and a speech bubble with a maximum of 160 characters per screen. The player moves to the next page by tapping on the screen. They can also click a back button to reread the previous screen. The type of introduction used in eQuoo is commonly known as game lore, a game-specific mythology, or the so-called backstory of the general narrative within a game. Lore in video games has been proven to motivate players to read and learn more, making it more likely that they will engage, read, and therefore learn the skills presented [52]. In eQuoo, Lodestars travel through time and space to fight against the Quavering by growing their inner light. The following is the onboarding text:

For centuries, the Lodestars have watched over this world, and they would love for YOU to join them. Get ready for the ultimate adventure.

You’ll journey to different times in history, become friends with people from all walks of life. You’ll learn skills that will set you on a path towards personal growth. And you’ll help to counter a massive threat.

This threat is called The Quavering. A force created from all the greed and negativity in the world. Whenever someone’s inner light grows dim, The Quavering grows stronger. You may have experienced this? If you join the Lodestars, you will help fight the battle against The Quavering.

So, here you are. You have been given a chance to grow your inner light. And to shine that light on others. Welcome... to the Lodestars.

The game consists of multiple books of different genres (eg, fantasy, historical drama, and teen drama). Each book consists of 8 to 10 chapters where the player can learn and repractice up to 4 skills. Before each new chapter of a book in the game, they are led through a gamified skill tutorial by the game’s guide, Joy, who is introduced to have been a player themselves and is now a Lodestar, thanks to having completed the game. Once they have successfully learned the skills, they continue into the book where they—playing themselves via an avatar that they customized at the beginning of their journey—meet the characters of the game and are thrown into various situations where they need to correctly use the psychological skills that they learned in the tutorial. To facilitate between-session learning and incorporation of the necessary skills, users must wait 7 days until the next chapter unlocks. This allows them to practice the skills in real-life settings, which has a positive impact on the therapeutic outcomes [53]. The weekly lock is also to protect players from addictive patterns and not flood them with too much information that they have retained after only one session.

Of the 18 gamification elements named by Cheng et al [54], 11 have been incorporated into eQuoo:

1. Levels
2. Points—in the form of gem shards
3. Rewards—in the form of unlocking levels and completing gems
4. Narratives
5. Personalisation—in the form of the story choices
6. Customisation—in the form of the avatar
7. Mini games
8. Quests and challenges—in the form of stories
9. Badges—in the form of personality types
10. Artificial assistance—in the form of the guide, Joy
11. Unlockable content—in the form of a free trial

The intervention group was instructed to download eQuoo via the Apple or Google Play Store and install it on their digital device. Participants were informed at the start of the study that they could stop using the app at any time. After starting the game, the player is introduced to the game’s lore. Participants are asked to design an avatar that resembles themselves as a virtual person in the game. The content of the application is divided into multiple multigenre stories that consist of 8 to 10 chapters presented as levels. In the chapters, users are first presented with 1 to 4 lessons that teach them psychological concepts, such as emotional bids [55], generalization [56], catastrophization [57], beliefs [58], and 52 psychological skills commonly used in therapeutic sessions for anxiety and depression (as well as prevention programs designed to increase resilience). After each lesson, the players can test their mastery of skills using a simple multiple-choice scenario. They are then either debriefed on why their choice was not the most beneficial (and are invited to choose another answer) or allowed to enter an interactive adventure story where they play themselves while practicing the skills in a low-cost environment. A low-cost environment means that the failure to succeed comes at a low cost, such as having to replay a level. All prompts during the psychoeducational part of the game to check whether the player has understood the skill are divided into 3 responses: (1) beneficial—the skill has been implemented by the player in a way that is beneficial for the player’s mental health; (2) neutral—the skill has been ignored and not used, and the counter indication was not chosen; and (3) unbeneficial—the player chose an answer that is considered unbeneficial for the player’s mental health.

The onboarding of the game consists of 3 levels of introduction to the lore; the building of the avatar; and the first in-app baseline assessment of the RRM, GAD-7, and PHQ-8 in the form of a pop-up chatbot where the player can chat with the game’s guide, Joy, and fill out the questionnaires. After acquiring their second skill, they play the first chapter of the first story and hit the level lock until the next week. This ensures that they play only 1 level per week for 5 weeks of the clinical trial. Each survey includes a question that players can answer only if they have completed the level for the week. Biweekly in-app nudges and weekly emails pull the participants back into the game.

#### Active Control Group Intervention (Sanvello)

Participants in the active control group were instructed to download an evidence-based mental health app called *Sanvello*, which was used as treatment-as-usual or active control group. It was chosen specifically to explore the secondary hypothesis of attrition, as the app has already been tested in another RCT [59] and is associated with a reduction in depression [60] and anxiety [61]. Sanvello is based on CBT and includes psychoeducation, CBT exercises, notifications, and a diary. It includes free access to multiple modules, which was sufficient to cover the 5-week trial. We requested that the participants use it for a minimum of 10 minutes per week. Participants were informed at the start of the study that they could stop using the app at any time.

#### Waitlist Control Group

The waitlist group received no intervention but completed the questionnaires at the same time points as the control and intervention groups. After completing the trial, they were debriefed on the results and provided with a link to both the eQuoo and the Sanvello apps. The study information was available via the link.

### Data Collection

Participants were reminded via weekly emails to complete the questionnaires at t1 to t5. In addition to the questionnaires, the participants in the eQuoo group were asked a question to prove that they had completed their level, and the participants in the active control group were asked if they had spent 10 minutes on the Sanvello app. Data were collected using LimeSurvey, a widely used secure open-source tool.

### Statistical Analysis

Differences in participant characteristics between each intervention arm were first compared using chi-square tests of independence (for categorical variables) and one-way ANOVAs (for continuous variables).

Attrition rates and the number of participants who completed the levels at all time points were compared across groups. Attrition was defined as not completing the assessments past t0. This is consistent with previous studies that defined attrition as the failure to complete the study protocol associated with the intervention [22]. To compare the likelihood of attrition between intervention arms, logistic regression models were constructed, with both unadjusted and adjusted (for sex, age, living situation, and baseline measures) odds ratios (ORs) and 95% CIs reported.

A standardized outcome was then created using the last available measure, even if it was only the participant’s baseline, and this was carried forward (*intention to treat*) [62]. Initially, paired sample 2-tailed *t* tests were conducted within each intervention arm to assess statistically significant changes in the primary and secondary outcomes. Pre-post effect sizes (Cohen *d*) were calculated using 0.2, 0.5, and 0.8, which were used as thresholds to signify small, medium, and large effects, respectively. Differences between intervention arms in end point (t5) scores were explored using linear regression models (for each outcome) with a baseline measure, and age, sex, living situation, and study level (ie, undergraduate or postgraduate) were entered as covariates. Adjusted end point means were estimated, and the magnitude of between-group differences was explored by calculating Cohen *d*.

To assess differences between interventions over time points, initial analyses used RM-ANOVAs with time (6 levels) entered alongside the intervention group (3 levels). To assess the impact of listwise deletion on these models, further analysis was conducted using mixed effects models exploring changes in the primary and secondary outcomes over time, entering the intervention arm as an independent variable and age, sex, and living situation as covariates. These mixed effects models, using restricted maximum likelihood estimation, used all available data at each time point. The survey was programmed so that all fields were mandatory, that is, participants either filled out the entire questionnaire or did not participate. The data were published using the Open Science Framework [63].

## Results

### Attrition and Characteristics by Group

**Figure 1:**
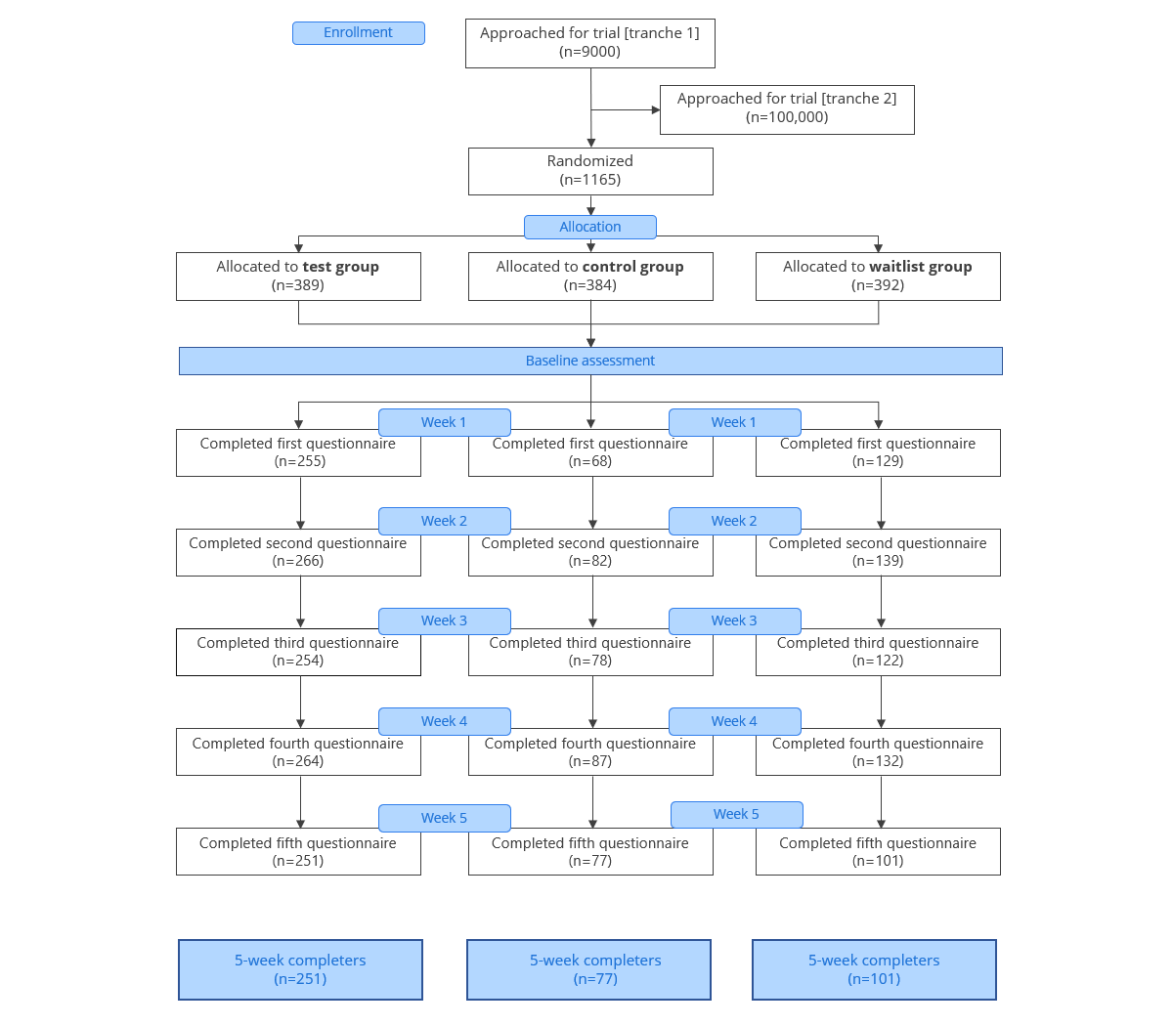
Study procedure and number of completers at each stage of the assessment.

Of the 1167 individuals who were recruited for the study, 2 individuals reported being aged <18 years and were excluded; therefore, the final sample comprised 1165 participants. These individuals were then randomly allocated to the study groups: 389 (33.39%) were placed in the eQuoo group, 384 (32.96%) were placed in the Sanvello group, and 392 (33.65%) were placed in the waitlist group. Figure 1 shows the participant flow diagram and the proportion of participants providing data at each time point. All participants (except 1 individual in the eQuoo group) completed baseline (t0) measures, and end point (t5) measures were available for 251 (64.5%) out of 389 participants in the eQuoo group, 77 (20%) out of 384 participants in the Sanvello group, and 101 (25.8%) out of 392 participants in the waitlist group. Of the eQuoo participants, 349 completed at least one measure after baseline (but potentially not t5), and 123 of the Sanvello participants completed at least one nonbaseline measure, as did 211 of the waitlist group.

The likelihood of attrition (defined as completing only the baseline measures) was compared for the intervention conditions. Although only 10.3% (40/389) of the participants in the eQuoo group met the criteria for attrition, the rates for the Sanvello and waitlist groups were 67.9% (261/384) and 46.2% (181/392), respectively. The odds of attrition were significantly higher in the Sanvello group than in the eQuoo group (OR 18.51, 95% CI 12.52-27.38) as well as in the waitlist group (OR 7.48, 95% CI 5.10-10.97). After adjusting for age, sex, living situation, study level, and all 3 baseline measures, Sanvello was compared with eQuoo (OR 19.27, 95% CI 12.91-28.77), as was the waitlist (OR 7.34, 95% CI 4.96-10.85).

Table 1 presents differences in participant characteristics between intervention arms. The comparative statistics suggest the groups were balanced in terms of age, sex, and baseline resilience scores but not living situation, study level, or initial depression and generalized anxiety symptom scores.

**Table 1:** Participant characteristics between the intervention arms.

Characteristic		Total (n=1165)	eQuoo (n=389)	Sanvello (n=384)	Waitlist (n=392)	*P* value	
**Wave, n (%)**							.91
	Wave 1	443 (38)	145 (37.3)	149 (38.8)	149 (38)		
	Wave 2	722 (62)	244 (62.7)	235 (61.2)	243 (62)		
**Sex, n (%)**							.43
	Female	891 (76.5)	285 (73.3)	297 (77.3)	309 (78.8)		
	Male	251 (21.5)	95 (24.4)	79 (20.6)	77 (19.6)		
	Other	18 (1.5)	6 (1.5)	6 (1.6)	6 (1.5)		
	Missing	5 (0.4)	3 (0.8)	2 (0.5)	0 (0)		
**Living situation, n (%)**							.03
	Close family or relatives	471 (40.4)	142 (36.5)	170 (44.3)	159 (40.6)		
	Student apartment	234 (20.1)	83 (21.3)	75 (19.5)	76 (19.4)		
	Own apartment	329 (28.2)	126 (32.4)	102 (26.6)	101 (25.8)		
	Other	124 (10.6)	36 (9.3)	32 (8.3)	56 (14.3)		
	Missing	7 (.6)	2 (0.5)	5 (1.3)	0 (0)		
**Level of education, n (%)**							.02
	Undergraduate	717 (61.5)	217 (55.8)	246 (64.1)	254 (64.8)		
	Postgraduate	446 (38.3)	170 (43.7)	138 (35.9)	138 (35.2)		

### Effects of Treatment on Primary Outcome (Resilience)

Pre- and posttest scores for resilience are presented in Table 2. There was a significant improvement (medium effect size) in resilience among eQuoo participants (t_387_=18.35; *P*<.001; Cohen *d*=0.58) but not among Sanvello or waitlist participants (*P*=.10 and .82, respectively).

Differences in end point scores were then compared using linear regression models, controlling for baseline resilience score, age, sex, living situation, and study level. Significantly higher resilience scores were observed in the eQuoo condition compared with Sanvello (*b*=−4.41, 95% CI −5.09 to −3.74; *P*<.001) and in eQuoo compared with the waitlist condition (*b*=−4.78, 95% CI −5.46 to −4.10; *P*<.001).

**Figure 2:**
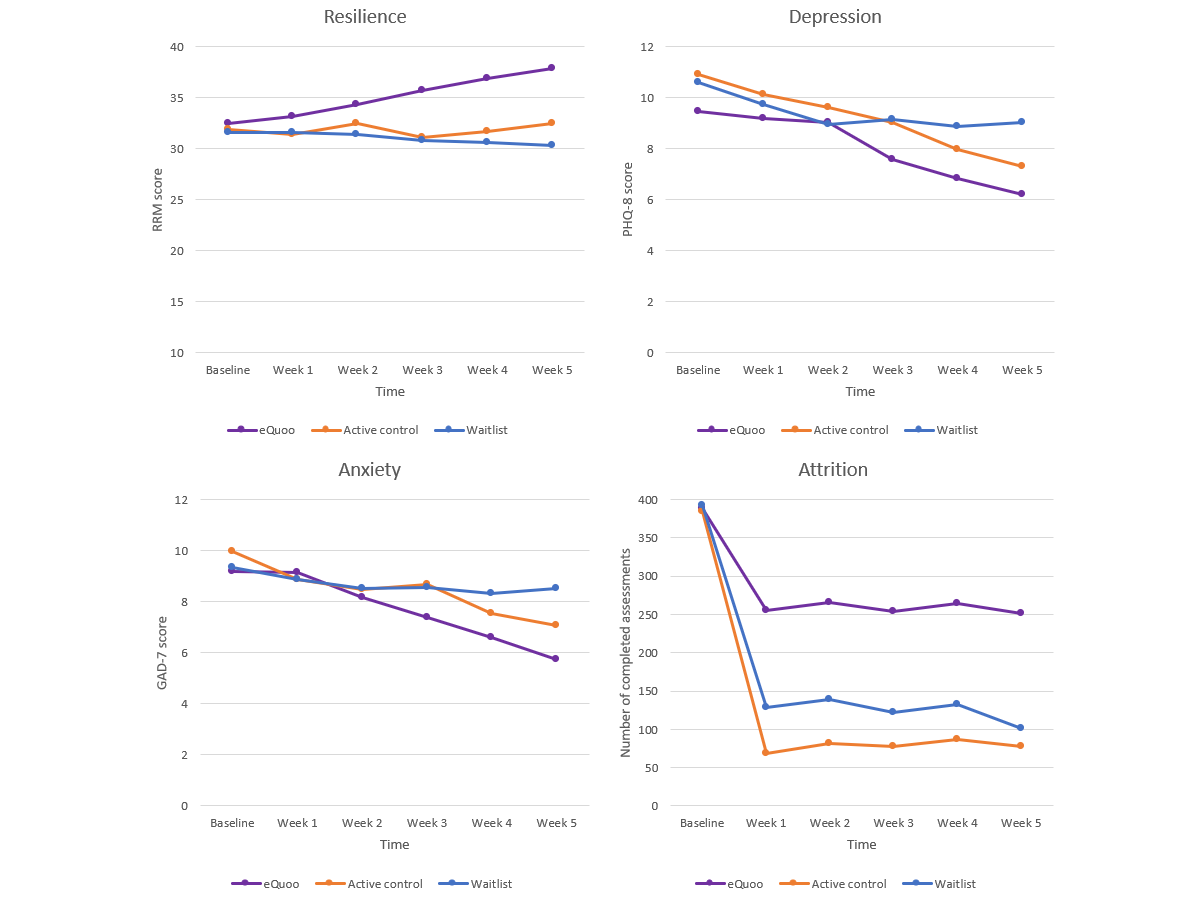
Average (unadjusted) weekly outcome scores by week (95% CIs) for resilience, depression, and anxiety. All available scores are included. GAD-7: generalized anxiety disorder–7; PHQ-8: Patient Health Questionnaire–8; RRM: Rugged Resilience Measure.

RM-ANOVA models were then used to compare resilience scores at each time point between the intervention arms, and mixed effects models were constructed to explore the change between the intervention arms. A total of 174 participants (n=78, 44.8% eQuoo; n=37, 21.3% Sanvello; and n=59, 33.9% waitlist) completed the measures at all time points and were included in the RM-ANOVA. A significant effect was observed for time (*F*_5,855_=18.51; *P*<.001), intervention arm (*F*_2,171_=8.68; *P*<.001), and intervention interaction (*F*_10,855_=9.69; *P*<.001). Mixed effects models, including age, sex, living situation, and study level as covariates led to a significant intervention-in-time interaction: scores were significantly lower over time for participants in the Sanvello arm (*b*=−0.86, 95% CI −1.06 to −0.66; *P*<.001) and waitlist arm (*b*=−1.25, 95% CI 1.42 to −1.08; *P*<.001), compared with the eQuoo arm (Figure 2).

**Table 2:** Pre- and postoutcome scores between intervention arms.

Outcome or intervention		Sample, n (%)	Baseline, mean (SD)	End point^a^, mean (SD)	*t* test (*df*)	*P* value
**Resilience**						
	eQuoo^b^	388 (99.74)	32.47 (8.35)	37.88 (7.17)	−18.35 (387)	<.001
	Sanvello	384 (100)	31.86 (7.66)	32.42 (7.94)	−1.65 (383)	.10
	Waitlist	392 (100)	31.60 (8.67)	30.34 (9.01)	0.22 (391)	.82
**Anxiety**						
	eQuoo	388 (99.74)	9.17 (6.91)	5.75 (4.26)	12.09 (387)	<.001
	Sanvello	384 (100)	9.98 (5.62)	7.06 (5.49)	5.77 (383)	<.001
	Waitlist	392 (100)	9.33 (5.68)	8.51 (5.82)	1.97 (391)	.049
**Depression**						
	eQuoo	388 (99.74)	9.44 (5.61)	6.18 (4.59)	20.91 (387)	<.001
	Sanvello	384 (100)	10.89 (5.61)	7.30 (5.39)	5.45 (383)	<.001
	Waitlist	392 (100)	10.59 (5.78)	9.02 (5.75)	3.45 (391)	.001

^a^End point scores included last observation carried forward for participants who did not complete an end point measure.

^b^One individual did not complete a baseline assessment.

### Effects of Treatment on Secondary Outcomes

#### Generalized Anxiety

In the pre-post analysis, GAD-7 scores significantly decreased within all 3 conditions (eQuoo: t_386_=22.86, *P*<.001; Cohen *d*=0.60; Sanvello: t_347_=5.77, *P*<.001; Cohen *d*=0.16; and waitlist: t_391_=1.97, *P*=.049; Cohen *d*=0.08; Table 2). Linear regression models demonstrated significantly lower GAD-7 end point scores in the eQuoo condition compared with the Sanvello (*b*=2.28, 95% CI 1.83-2.73; *P*<.001) and waitlist conditions (*b*=2.54, 95% CI 2.08-2.99; *P*<.001).

The RM-ANOVAs, including only those participants who completed every time point, showed that although there was a significant main effect of time (*F*_5, 855_=29.337; *P*<.001), there was no main effect of condition (*P*=.35) or a condition-by-time interaction (*P*=.10). Further analysis performed using mixed effects models (which included the covariates listed earlier) indicated a significant time-by-condition interaction; over time, GAD-7 scores were lower in the eQuoo group compared with the waitlist group (*b*=0.56, 95% CI 0.40-0.71; *P*<.001). The difference between the eQuoo and Sanvello conditions was not significant (*b*=0.12, 95% CI 0.06-0.30; *P*=.20; Figure 2).

#### Depression

In the pre-post analysis, the PHQ-8 scores significantly decreased in all 3 groups (*P*≤.001). However, although changes in depression scores produced a medium effect (Cohen *d*=0.58), they were small for the Sanvello and waitlist groups (Cohen *d*=0.14 and 0.13, respectively; Table 2).

As with the GAD-7 results, the linear regression demonstrated significantly lower PHQ-8 end point scores in the eQuoo condition compared with the Sanvello group (*b*=2.47, 95% CI 2.91-2.93; *P*<.001) and in the eQuoo condition compared with the waitlist group (*b*=2.46, 95% CI 2.00-2.91; *P*=.15).

The RM-ANOVAs for PHQ-8 scores, including only those individuals who completed every time point, indicated that although there was a significant main effect of time (*F*_5,855_=55.392; *P*<.001), there was no main effect of condition (*P*=.22) or a condition-by-time interaction (*P*=.11). The mixed effects models (which included the covariates listed earlier) indicated a significant time-by-condition interaction, showing that over time, PHQ-8 scores remained lower for the eQuoo group compared with waitlist group (*b*=0.40, 95% CI 0.25-0.55; *P*<.001). The difference between the eQuoo and Sanvello conditions was not significant (*b*=0.09, 95% CI −0.09 to 0.27; *P*=.31).

## Discussion

### Principal Findings

The results suggest that using the gamified mental health app, eQuoo is an effective pathway for improving mental health and resilience. In particular, the use of eQuoo was related to increased resilience scores over time; this was not the case for the nongamified or waitlist groups. Participants using eQuoo also reported lower depression and anxiety scores at the end of the intervention compared with the other 2 groups and were significantly less likely to drop out of treatment. The findings indicated that both apps were effective in reducing anxiety and depression symptoms, although the larger effect sizes associated with the improvements suggest that eQuoo was more effective in this regard.

Mental health issues are pervasive and particularly impactful in student populations [64,65]. Unfortunately, the gap between mental health treatment needs and access to care continues to grow [66,67]. Although DHIs have been identified as meaningful alternatives to face-to-face therapy in student populations [9,11], they have significant shortcomings [68-70] in terms of motivation, interest, and engagement [69]. As the prevalence of mental health issues [69] and the demand for digital mental health solutions, such as telehealth and internet therapy [71] have increased, research investigating digital, app-based interventions is especially timely. This study contributes to the quest for solutions by presenting eQuoo as a gamified DHI that can improve mental health by reducing depression and anxiety symptoms while building resilience and compares favorably with a well-established, nongamified mental health app.

### Implications

Gamification using mobile mental health apps has been suggested as a way to increase engagement in mental health services [72-74]. This study supports this suggestion by highlighting the benefits of DHIs incorporating gamification and resilience training (particularly when eQuoo was used). Although further research that distinguishes the effects of gamification and includes more diverse populations is necessary, the results of this study suggest that mobile mental health apps can assist in mental health treatment and resilience building.

Furthermore, our findings are particularly relevant, given the current events. Global factors, such as the 2019 COVID-19 pandemic and the accompanying restrictions, have been linked to an increased prevalence of mental health issues and difficulties in accessing support [75]. According to a recent World Health Organization survey, the pandemic has disrupted critical mental health services in 93% of countries worldwide while increasing demand for them [50]. Our findings suggest that DHIs may be a meaningful option in lieu of traditional mental health services for those seeking help.

The finding that resilience scores improved for eQuoo users is also promising, as this suggests that the impact and lingering effects of future adversity on mental health may be less for those with higher scores [74,76], among other benefits [77]. In other words, the inclusion of resilience training elements in DHIs such as eQuoo can facilitate a reduction in psychopathology symptoms such as depression and anxiety but may also serve a protective function against these symptoms for users when encountering difficulties later on. This would be especially important for young adults who experience many stressful transitions in different life domains [78]. Further longitudinal research is needed to explore this empirically, capturing both the long-term impact of eQuoo and the impact of subsequent stressors.

With continued research, DHIs that incorporate gamification and resilience training may play a pivotal role in improving access to mental health services and preventing mental health issues. They could be integrated into standard face-to-face treatment as part of the client’s homework, offered as a pretreatment option, or used as a mental health resource for all college students as part of an intervention and prevention strategy.

### Strengths and Limitations

This study has several strengths. It is one of only a few follow-up RCTs involving a gamified mental health app [51]. Its large participant pool enables calculations to be performed with a large power size, thus ensuring the reliability of the outcome. This study is based on past research on the efficacy of the same gamified app that has been tested and shown to increase resilience and positive relations with others and decrease anxiety symptoms in a very different sample (employed adults) [51]. Given this (and the similarities in findings between the present and previous studies), the benefits of eQuoo may be generalized to different populations. Finally, the study included 3 treatment arms, including a treatment-as-usual active control condition using a nongamified app that has been tested [59] and shown to be efficacious [59,61]. Including this treatment-as-usual active control condition allowed the gamified app and nongamified app to be usefully compared. In addition, unlike previous studies, we have explicitly reported on dropout and nonuse using engagement data.

This study has some limitations. Although including a treatment-as-usual active control condition was important to evaluate eQuoo, the 2 apps were not structurally equivalent interventions. Ordinarily, structurally equivalent interventions are identical in terms of the number and duration of sessions, settings (group vs individual), level of therapists’ experience, and adaptability of the therapy to the client [79]. The 2 apps used in this study have different purposes and use different interventions. Although the eQuoo is designed to teach psychological skills to decrease depression and anxiety and increase resilience, and many of these skills are based on CBT principles that overlap with the structure of Sanvello, the latter relies exclusively on CBT psychoeducation and exercises [59]. In other words, there were meaningful differences between the 2 apps, in addition to gamification. A meta-analysis comparing in-person psychotherapies found no differences between programs when they had structural equivalence [79]. Thus, it may be that the differences between eQuoo and Sanvello were not solely caused by gamification. In light of this, future studies might compare structurally equivalent gamified and nongamified apps, although a robust comparison would involve a version of the experimental app with gamification features removed, to ensure that there were no other explanatory variables, such as the look or interface of the app.

Another limitation of this study is that it did not record the duration of participants’ use. Hence, it was not possible to examine whether the level of use was linked to attrition, an important phenomenon discussed in eHealth research [28]. In other words, although the attrition among participants using the gamified app was lower than that in the nongamified app and waitlist groups, it was not possible to examine the influence of active engagement. This could have been accomplished by including time-of-use data from the apps.

A further limitation was the lack of follow-up; therefore, we could not draw conclusions about the stability of the observed effects. As such, future researchers should investigate the impact of gamification on nonuse attrition, engagement, long-term attrition, and mental health.

Similarly, it was unclear which aspects of the intervention resulted in the observed effects. As the eQuoo was created as a skills training app, it may have been that the development of resilience and reduction in mental health symptomatology was the result of skills acquisition. However, specific skill acquisition was not examined. Studies in which individual skills were trained were excluded to investigate the mechanisms of the observed effects. Future researchers might, therefore, want to include pre- and postmeasures of trained skills.

The participants in this study were self-selecting; therefore, a self-selection bias may have influenced our findings. In addition, the study was fully reliant on self-report, which may have also affected the results through both response bias [80] and shared variance [81].

Given these sample-specific and method-specific limitations, future researchers might want to investigate the effects of gamification using more diverse samples and additional measures of efficacy, such as sleep duration and quality (objective measures related to depression) and clinical interviews. In addition, the authors are aware that using the traditional “gold standard” of RCTs may not be the best method for evaluating the effectiveness of an app such as eQuoo. Such studies can take years from inception to publication, and developers will have real-time feedback and data that would influence app improvements more quickly than they could be tested [82]. The authors are investigating current research possibilities that would help address this issue and will be implementing them in the coming years while documenting the results for peer review.

### Conclusions

This study aimed to investigate the effect of the gamified mobile mental health game eQuoo on levels of resilience, anxiety, depression, and attrition (one of digital mental health’s Achilles heels [83]) in a student population. Compared with the active control and waitlist groups, we found a significant increase in resilience scores, a decrease in depression and anxiety scores, and a significantly lower attrition rate. The results suggest that eQuoo is an engaging and effective means to support students’ mental health and build their resilience.

## Appendix

Multimedia Appendix 1 CONSORT-EHEALTH checklist (V 1.6.1).

## Abbreviations

CBT: cognitive behavioral therapy
DHI: digital health intervention
GAD-7: generalized anxiety disorder–7
OR: odds ratio
PHQ-8: Patient Health Questionnaire–8
RCT: randomized controlled trial
RM: repeated measures
RRM: Rugged Resilience Measure
